# Synthesis and Properties of Bio-Based Polycarbonates Containing Silicone Blocks

**DOI:** 10.3390/polym16101318

**Published:** 2024-05-08

**Authors:** Mengjuan Liu, Hui Wang, Wei Fang, Tao Lu, Jinsen Wang, Guozhang Wu

**Affiliations:** Shanghai Key Laboratory of Advanced Polymeric Materials, School of Materials Science & Engineering, East China University of Science & Technology, Shanghai 200237, China; 17767145848@163.com (M.L.); y30220861@mail.ecust.edu.cn (H.W.); y82210335@mail.ecust.edu.cn (W.F.); y30210811@mail.ecust.edu.cn (T.L.); y82230330@mail.ecust.edu.cn (J.W.)

**Keywords:** isosorbide, polycarbonate, silicone, conversion, compatibility

## Abstract

This study aims to investigate the effects of different hydroxy-terminated silicones on the properties of polycarbonate-silicone copolymers (ICS-PC) by introducing flexible and hydrophobic silicone into isosorbide-based polycarbonate through melt transesterification- polycondensation method. Through compatibility and transesterification experiments, it is confirmed that the alcohol-hydroxyl polydimethylsiloxane (*a*-PDMS) has higher reactivity and silicone conversion than the phenol-hydroxyl polydimethylsiloxane (*p*-PDMS), but the conversion does not exceed 81%. Polyether-modified silicone (PEMS) exhibits better compatibility and higher reactivity, thus resulting in higher conversion that can reach 86%. Effects of the type and content of silicone on the glass transition temperature (*T*_g_), optical transparency, saturated water absorption, and mechanical strength of ICS-PCs were also discussed. It is found that *p*-PDMS has higher *T*_g_, hydrophobicity, and mechanical strength with similar silicone content, but the total transmittance does not exceed 60%. In contrast, the PEMS system exhibits better optical transparency due to its improved compatibility with the PC matrix, with a total transmittance of up to 73%, *T*_g_ exceeding 150 °C while maintaining excellent flexibility and hydrophobicity. These results are helpful to further improve the comprehensive properties of bio-based polycarbonates.

## 1. Introduction

The traditional bisphenol A polycarbonate (BPA-PC) is widely applied in various fields such as electronics, the automotive industry, protective equipment, and aerospace due to its excellent comprehensive properties, including impact toughness, heat resistance, and optical transparency [1,2,3]. However, BPA-PC has been banned from food packaging, medical equipment, and other fields because its monomer, BPA, derives from non-renewable fossil resources and may endanger human health [4,5,6]. Isosorbide (ISB) is a bio-based material derived from glucose. It can be obtained by enzymatic hydrolysis of biomass sources such as starch and cellulose into glucose, followed by hydrogenation and dehydration reactions [7]. ISB is considered a promising bio-based material due to its rigidity, chirality, and non-toxic, and it is widely used to synthesize bio-based polycarbonate, polyurethane, polyamide, and other functional polymer materials [8,9,10,11]. Biobased polycarbonate can be used to manufacture car body parts, interiors, and other plastic components such as door panels, dashboards, and seats. Its lightweight properties contribute to improving fuel efficiency in cars while reducing dependence on fossil resources. However, the performance stability and cost-effectiveness of biobased polycarbonate still need to be further improved [12]. Moreover, the homopolymer of ISB is rigid, highly viscous, and difficult to process. Therefore, it is generally copolymerized with the flexible monomer 1,4-cyclohexanedimethanol (CHDM) to prepare bio-based polycarbonate (I*c*C-PC). I*c*C-PC exhibits superior optical transparency, UV resistance, low birefringence, and higher surface hardness than BPA-PC due to the absence of benzene rings in the main chain [13,14,15,16,17].

However, ISB is prone to absorbing water, and the glass transition temperature (*T*_g_) of the polymer decreases significantly after copolymerization with flexible monomer CHDM. Consequently, the I*c*C-PC system suffers from insufficient heat resistance, water sensitivity, and poor notch impact toughness, which limits its widespread application [18,19]. Thus, I*c*C-PC still requires further modification. Silicone (PDMS) is safe and non-toxic, with low surface tension and excellent thermal stability. Its flexible silicone main chain is arranged in a helical structure with hydrophobic methyl groups attached to the side chains, which can improve the melt flowability and hydrophobicity of the polymer. Importantly, it is possible to significantly increase *T*_g_ while maintaining the great toughness of the polymer by increasing the content of ISB, reducing the amount of CHDM, and introducing a certain amount of PDMS [20,21,22,23].

Silicone-modified polycarbonates are mainly modified by blending and copolymerization. Zhang et al. [24] directly blended PDMS with BPA-PC, resulting in physically blended PC/PDMS with island structures whose phase size was uncontrolled. This not only compromised the mechanical properties and optical transparency of the blend but also led to surface enrichment of low-surface-energy siloxane. Zhou et al. [25] studied PDMS modification of BPA-PC through reactive blending, and the size of PDMS phases decreased significantly from 15~20 μm in physical blends to 0.2~0.9 μm, but still an order of magnitude larger than copolymer systems (15~25 nm). Therefore, preparing PC-PDMS copolymers by chemically copolymerizing PDMS with other reactive monomers can effectively improve the compatibility between PC and PDMS. PC-PDMS copolymers are typically synthesized by the interfacial polycondensation method [26], but ISB is soluble in water, making it impossible to use the interfacial polycondensation method with dichloromethane and water as solvents. Accordingly, this study proposes a green, solvent-free melt transesterification-polycondensation method to synthesize PC-PDMS copolymers.

The key to preparing PC-PDMS copolymers through the melt transesterification-poly-condensation method is to increase the conversion rate of PDMS. There are three possible reasons for the low conversion rate of PDMS. Firstly, the differing reactivity of individual monomers is compounded by the high molecular weight and poor diffusion ability of PDMS. Secondly, significant differences in solubility parameters between individual monomers (*δ*_PC_ = 9.5 cal^1/2^cm^−3/2^ vs. *δ*_PDMS_ = 7.3 cal^1/2^cm^−3/2^) [27], rendering them thermodynamically incompatible. Thirdly, PDMS can undergo chain scission under high temperature, vacuum, or strong alkaline catalysis, resulting in the shortening of siloxane chain length and the residue of cyclic siloxanes [28,29]. Jiyunia et al. [30] used phenoxy-terminated PDMS, while Annett et al. [31] used BPA-terminated PDMS to balance the reactivity of each monomer yet observed no significant improvement in conversion rates. Koenig et al. [32] conducted transesterification reactions using BPA-PC oligomers and phenol-hydroxyl silicone. They found that the resulting product exhibited a distinct two-phase system when dissolved in chloroform, indicating that the conversion rate of PDMS had not increased. Zhou et al. [33] investigated the process of PDMS with different chain lengths participating in transesterification. They found that as PDMS chain length increased, the difficulty of miscibility between PDMS and DPC increased. Compared to PDMS with shorter chains, longer-chain PDMS required a higher critical level of transesterification for dissolution in DPC. Thus, screening appropriate silicones and increasing the compatibility of the reaction system are required to improve the conversion rate of silicones and enhance the comprehensive performance of copolymers.

To enhance the compatibility of monomers and prepare high-performance polycarbonate-silicone copolymers (ICS-PC), this study synthesized a polyether-modified silicone (PEMS) and synthesized copolymers by melt transesterification-polycondensation method. The compatibility of phenol-hydroxyl polydimethylsiloxane (*p*-PDMS), alcohol-hydroxyl polydimethylsiloxane (*a*-PDMS), and PEMS in the polymerization system and their effects on the transesterification reaction rate and conversion were discussed. Additionally, the thermal resistance, optical transmittance, hydrophobicity, and mechanical properties of copolymers were also examined to provide insights for preparing high-quality polycarbonates.

## 2. Materials and Methods

### 2.1. Materials

Isosorbide (ISB; 99.0%), 1,4-cyclohexanedimethanol (CHDM; 99.0%), and diphenyl carbonate (DPC; 99.0%) were provided by Ningbo Dafeng Jiangning New Materials Co., Ltd. (Ningbo, China). Phenol-hydroxyl silicone (*p*-PDMS; *M*_n_ = 2500 g/mol) and alcohol-hydroxyl silicone (*a*-PDMS; *M*_n_ = 2570 g/mol) were purchased from Anhui Ayota Co., Ltd. (Bengbu, China). Polyether silicone (PEMS; *M*_n_ = 3500 g/mol) was self-made by the laboratory. Sodium hydroxide (NaOH, 99%) was purchased from Shanghai Aladdin Biochemical Technology Co., Ltd. (Shanghai, China). Deuterated chloroform (CDCl_3_; 99.8%) was purchased from Beijing Brilliant Reagent Co., Ltd. (Beijing, China). Dichloromethane (CH_2_Cl_2_; 99.5%), chloroform (CHCl_3_; 99.0%), acetonitrile (C_2_H_3_N) and absolute ethanol (CH_3_CH_2_OH; 99.7%) were purchased from Shanghai Titan Technology Co., Ltd. (Shanghai, China).

### 2.2. Synthesis of PEMS

PEMS was synthesized by the polymerization of hydrogen-terminated silicone oil (PHMS) and allyl polyether glycol (APEG) through the hydrosilylation method without the use of solvents. APEG and PHMS were added to a three-necked flask in a molar ratio of 2.2, along with a catalyst of H_2_PtCl_6_·6H_2_O with a relative PHMS of 5 ppm. The reaction was maintained at a stirring speed of 200 to 250 rpm/min and reacted for 5 h at 110 °C under the nitrogen atmosphere. As the hydrosilylation reaction proceeded, the solution gradually changed from milky white to transparent, finally obtaining colorless and transparent PEMS.

### 2.3. Experiments on the Kinetics of Transesterification Reactions

Firstly, ISB and CHDM (ISB/CHDM = 90/10), DPC (DPC/Diols = 1.0), and 15 wt% silicone were added to a 100 mL four-necked flask. The flask was evacuated and refilled with dry nitrogen gas three times to remove air. Then, heat the oil bath to 180 °C, take the mixed sample as the starting sample, add 2 ppm NaOH catalyst immediately, and start timing. A small amount of sample was extracted from the reactor at set intervals. Each sample was sealed and stored in a refrigerator for analysis by SPD-20A high-performance liquid chromatography (HPLC). The melt transesterification reaction is a second-order reaction, and initially, only the forward ester exchange reaction between the diol monomer and DPC occurs. The transesterification reaction rate constant *k*′ can be represented by

*k*′t = 1/[A] − 1/[A]_0_ = [P]/([A]_0_^2^ − [A]_0_[P]) (1)

### 2.4. Synthesis of ICS-PC

**Scheme 1:**
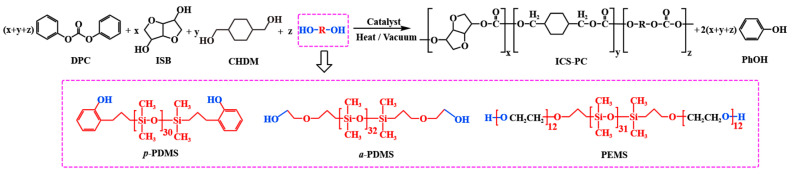
Chemical structure of monomers used for ICS-PC synthesis by the melt transesterification-polycondensation method.

The molecular structure of the monomers and the polymerization process are illustrated in Scheme 1. Initially, DPC, ISB, CHDM, silicone, and NaOH of 2 ppm to diols were added to a 100 mL three-necked flask. The molar ratio of DPC/diols was set at 1.01, and the ISB/CHDM molar ratio was set at 90/10. Under the N_2_ atmosphere, the mixture was heated to 160 °C and then started stirring. After 30 min, the temperature was increased to 180 °C and maintained for 1 h to make the transesterification reach equilibrium. After completing transesterification, the temperature was increased to 210 °C, and the pressure was reduced to 20 kPa for 30 min. Subsequently, the temperature was further increased to 230 °C, and the pressure was reduced to 12 kPa, holding for 20 min. Finally, the temperature was increased to 250 °C, and the pressure was reduced to 4 kPa, maintained for 10 min, then evacuated to 80 Pa for 10 min. N_2_ was then introduced into the reaction vessel to maintain atmospheric pressure for 30 min. The resulting 6~7 g product was dissolved in 100 mL of CHCl_3_ and precipitated using 500 mL of anhydrous ethanol. This dissolution-precipitation process is repeated thrice to remove unreacted silicone oil, other monomers, and oligomeric by-products. The precipitated product is concentrated by filtration, then vacuum dried at 80 °C for 24 h for structural and performance testing.

### 2.5. Preparation of Copolymer Films

Polycarbonate films were prepared by the solvent casting method. Initially, the samples were dried in an 80 °C oven for 12 h to remove moisture and dissolved in CHCl_3_ to make a solution with a mass fraction of about 5%. After using ultrasonic agitation for 10 min at 20 °C to remove bubbles from the solution, they were poured onto a flat dish and left at room temperature for 24 h to allow most of the solvent to evaporate. Finally, the samples were placed in a vacuum oven and gradually heated to 50 °C, 70 °C, and 90 °C, holding each temperature for 12 h to expel any residual solvent. The samples were then heated above *T*_g_ to remove thermal history, yielding PC films with a thickness of about 80 μm, suitable for subsequent performance testing.

### 2.6. Characterizations

^1^H-NMR spectroscopic measurements were conducted using an AVANCE 400 NMR spectrometer (Bruker Corporation, Karlsruhe, Germany). CDCl_3_ with TMS served as deuterium solvents. 

To determine the viscosity-average molecular weight (*M*_η_) at 25 ± 0.5 °C using a Ubbelohde viscometer, the product was dissolved in CHCl_3_ to prepare a solution with a concentration of 0.01 g/mL. The intrinsic viscosity [*η*] was calculated by the following formula:

(2) $$\eta_{\mathrm{sp}}=\frac{t}{t_{0}}-1\;$$

(3) $$\;\eta_{r}=t\;/t_{0}$$

(4) $$\left[\eta \right]=\sqrt{2\left(\eta_{\mathrm{sp}}-\ln\eta_{r}\right)\;/\;C}\;$$

where *C* is the concentration of the solution, *t*_0_ and *t* are the times when the pure solvent and the sample solution flow through two scales of the Ubbelohde viscometer, respectively. *η*_r_ is the relative viscosity, and *η*_sp_ is the specific viscosity. *M*_η_ of the polymer was calculated using the Mark–Houwink equation:

(5) $$\left[\eta \right]=KM_{\eta }^{\alpha }\;$$

where *K* is 0.0111 mL/g, and α is 0.82 [34].

Color difference (∆*C*) was measured with an ultraviolet spectrophotometer (UV; UV1900, Youke Instrument, Shanghai, China). A CHCl_3_ solution with a concentration of 0.01 g/mL was used, and ∆*C* was calculated by the following formula:

(6) $$\Delta C=\left(\;\left|\frac{1}{3}-\frac{T_{445}}{T}\right|+\left|\frac{1}{3}-\frac{T_{555}}{T}\right|+\left|\frac{1}{3}-\frac{T_{600}}{T}\right|\;\right)\times 100\%$$

where *T*_600_, *T*_555_, and *T*_445_ represent the transmittance of the solution relative to the pure solvent at wavelengths of 600, 555, and 445 nm, respectively. Furthermore, *T* = *T*_600_ + *T*_555_ + *T*_445_.

With air as the blank reference, test the light transmittance of the copolymer film in the visible light area (380~780 nm) and calculate the total transmittance *T*_total_ (%) by the following formula:

(7) $$T_{\mathrm{total}\;}\left(\lambda \right)=\frac{|\int_{380}^{780}T\left(\lambda )\;\right|}{780-380}\;$$

The glass transition temperature (*T*_g_) was measured by DSC (Discovery 25, TA Instruments, New Castle, DE, USA). The sample was first heated to 220 °C at the rate of 30 °C/min, stabilized for 2 min to eliminate the thermal history, and then dropped to 20 °C at the same rate. Finally, the temperature is raised to 250 °C at the rate of 10 °C/min. *T*_g_ was determined as the inflection point of the second heating curve.

The morphological changes of silicone dispersed in the system were characterized by an MDS-30 optical microscope (Olympus Corporation, Tokyo, Japan). Specifically, 5 wt% of silicone was added to the reaction system and uniformly dispersed by melting and stirring at 160 °C. We took a drop on the glass slide, gently pressed the sample to flow naturally, and observed the changes in two-phase morphology using a lens of 40 × 10.

The hydrophobicity of the copolymer films was measured using a contact angle measuring instrument (JC2000D20, Zhongchen Digital Technology Equipment Co., Ltd., Shanghai, China). A 0.2 μL droplet of pure water was dropped onto the surface of the sample using a micro syringe, and three measurements were taken at different positions on the same sample to obtain the average value.

The initial weight of the copolymer film was recorded after vacuum drying at 50 °C for 12 h. It was then soaked in water at 80 °C, and the surface moisture was wiped off and weighed at regular intervals. The water absorption rate was calculated based on the change in mass before and after water absorption.

The mechanical properties of the samples were tested using a universal testing machine (Instron 3365, Instron Corporation, Boston, MA, USA). The films were cut into strips with dimensions of 80 × 8 × 0.08 mm^3^ and tested at room temperature with a tensile rate of 10 mm/min. Five tests were performed for each sample, and the average value was calculated.

## 3. Results and Discussions

### 3.1. Compatibility Analysis of Silicone with Other Monomers

**Figure 1:**
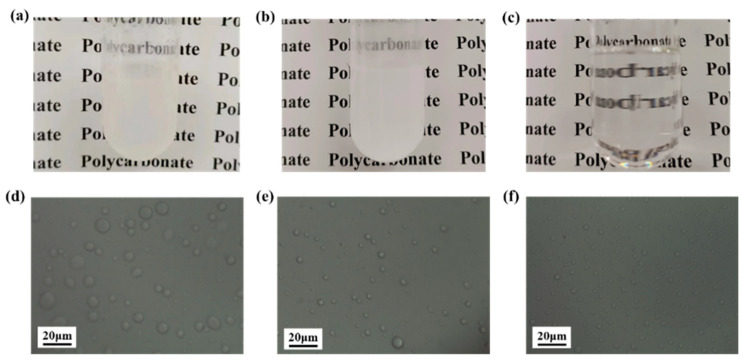
Optical clarity of monomer mixtures with 5wt% (**a**) *p*-PDMS, (**b**) *a*-PDMS, and (**c**) PEMS loading at 160 °C. Dispersion of (**d**) *p*-PDMS, (**e**) *a*-PDMS, and (**f**) PEMS in monomer mixtures.

Scheme 1 shows the molecular structures of all monomers in the reaction system. Among them, the three silicone lengths are 31 ± 1. Compared with a-PDMS, the number of oxyethylene units at both ends of PEMS has increased from 1 to 12, aiming to improve the compatibility of PEMS with ISB and CHDM. The set reaction system is as follows: the molar ratio of ISB/CHDM was set at 90/10, the molar ratio of DPC to diols was set at 1.01, and *p*-PDMS, *a*-PDMS, and PEMS of 5 wt% loading was added respectively. After melting at 160 °C, the reaction system was stirred by magnetic force for 5 min to ensure uniform mixing. Figure 1a–c shows photos of the appearance of the reaction systems. It is observed that the *p*-PDMS, *a*-PDMS, and PEMS systems exhibit light yellow turbidity, milky turbidity, and semi-transparency, respectively, indicating that the *p*-PDMS system has the worst compatibility and the PEMS system has the best compatibility.

Figure 1d–f shows the dispersion states of the silicones in the molten system at 160 °C observed under a microscope. The dispersed phase size of the *p*-PDMS system is 10 ± 5 μm, which is quite uneven; the phase size of *a*-PDMS is 5 ± 4 μm, and PEMS is more easily dispersed in the reaction system with a phase size reduced to 2 ± 2 μm. The microscopic dispersion states are consistent with the appearance results, indicating that the smaller and more uniform the dispersed phase size of silicone, the better the compatibility of the reaction system. This could be explained by the fact that PEMS contains polar and highly surface-active polyether segments, which makes PEMS contact more hydrophilic monomers such as ISB and CHDM, thus improving the compatibility of the system [35]. However, PEMS and the system still can not achieve molecular-level miscibility, retaining a certain phase size because of the lack of catalyst and no transesterification reaction occurred.

### 3.2. Effect of Silicones on Transesterification Reaction Rates

**Figure 2:**
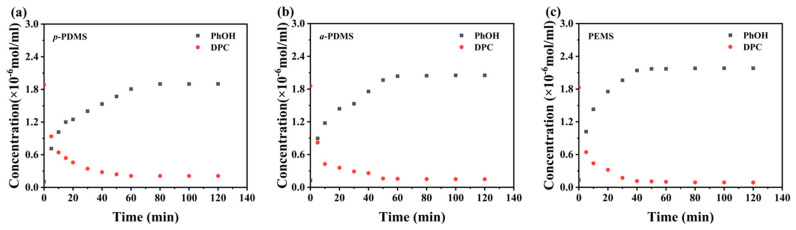
The concentration changes of PhOH and DPC during transesterification of DPC and diols at 180 °C in the system with 15 wt% (**a**) *p*-PDMS, (**b**) *a*-PDMS, and (**c**) PEMS loading.

Figure 2 shows the concentration change of PhOH and DPC during transesterification at 180 °C in the system with 15 wt% silicone loading when the molar ratio of ISB/CHDM was set at 90/10 and the molar ratio of DPC/diols was set at 1.0. It can be observed that the concentration of DPC gradually decreases with the extension of reaction time while the concentration of PhOH increases and tends to balance. Among them, after 120 min of transesterification reaction, in the *p*-PDMS system, the residual amount of unreacted DPC is more, and the transesterification equilibrium time exceeds 80 min. The *a*-PDMS system reaches transesterification equilibrium at about 60 min. In contrast, the transesterification reaction speed of the PEMS system is significantly accelerated, reaching transesterification equilibrium at around 40 min, and the residual amount of unreacted DPC is minimal.

**Figure 3:**
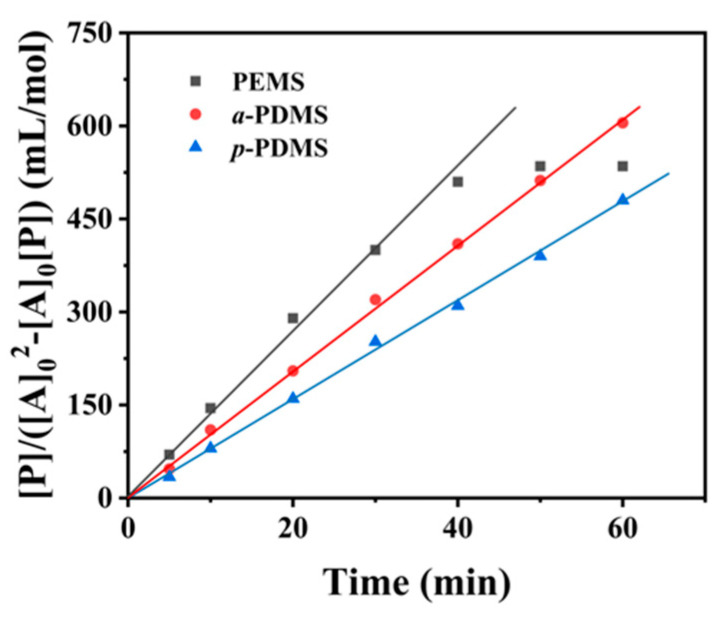
Determination of transesterification reaction rate constant for systems with different silicone types.

According to Formula (1), the transesterification reaction rate constant *k*′ can be obtained from the slope of the straight line in Figure 3. Table 1 lists the relevant data showing that the *k*′ of the PEMS system is the largest, indicating that the reactivity of PEMS is greater than that of *a*-PDMS and the reactivity of *p*-PDMS is the poorest. Because PEMS disperses better in the reaction system in the molten state, making more complete transesterification reactions at the silicone interface. The reactivity of *a*-PDMS is higher than that of *p*-PDMS, in addition to the slight difference in the size of the dispersed phase; the larger reason may be that the phenolic hydroxyl group exhibits steric hindrance due to the benzene ring, inhibiting the nucleophilic substitution reaction of oxygen anions with electrophilic groups, thus making the alcohol hydroxyl group more reactive than the phenolic hydroxyl group [36].

### 3.3. Analysis of Silicone Conversion

In the system where the molar ratio of ISB/CHDM was set at 90/10 and the molar ratio of DPC/diols was set at 1.01, different mass fractions of silicone were added to prepare various ICS-PC. Their basic properties are listed in Table 2. Additionally, I*c*C-PC is a commercial product with an ISB/CHDM molar ratio of 70/30.

**Figure 4:**
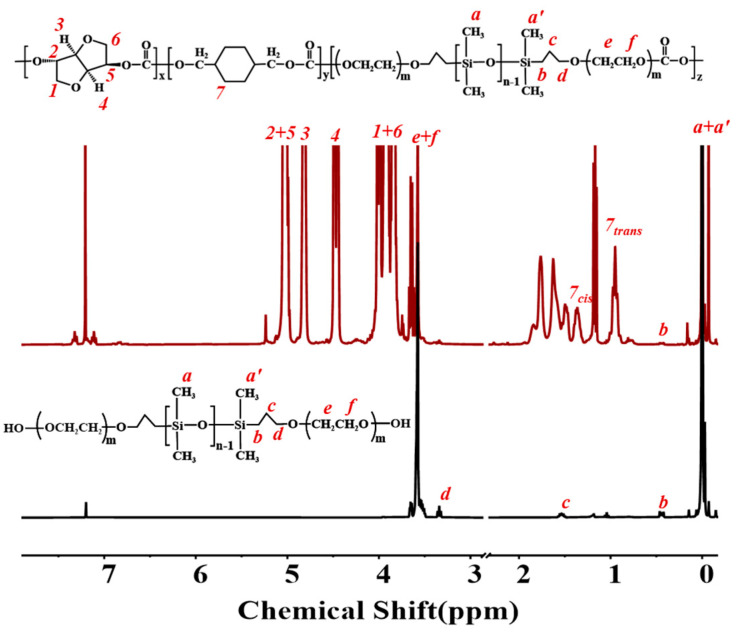
^1^H-NMR spectra of PEMS and ICS-PC containing PEMS.

The unreacted silicone monomers and oligomers can be effectively removed through repeated precipitation and washing three times. The ^1^H-NMR spectra of ICS-PC containing PEMS are shown in Figure 4, and the ^1^H-NMR spectra of copolymers containing *p*-PDMS and *a*-PDMS are shown in Figures S1 and S2. The block contents (*W*, wt%) and conversions (%) of silicones can be calculated using ^1^H-NMR. They were measured by the following:

(8) $$W=\frac{\frac{74\;\times \;I_{a+a^{\prime }}}{6}}{\frac{74\;\times \;I_{a+a^{\prime }}}{6}+I_{4}\;\times \;172+\frac{I_{7}}{4}\;\times \;170\;}\;\times \;100\%\;\;\;$$

(9) $$\mathrm{Conversion}=\frac{W}{W_{0}}\times 100\%\;$$

where *I*_x_ represents the integrated area of the corresponding proton peak in Figure 4. When x is *a+a*′, the corresponding peak position is −0.02 to 0.30 ppm, which is the proton peak of the Si-CH_3_ side chain in the silicone oxygen chain. Peak b corresponds to the proton peak of the hydrogen atom on the first Si-CH_2_ group connected to the terminal silicon atom observed at around 0.45 to 0.57 ppm. Peaks 7_cis_ observed at 1.29 to 1.43 ppm and 7_trans_ observed at 0.87 to 1.08 ppm correspond to the two cis-OH and two trans-OH proton peaks of CHDM, respectively. Peak 4 represents an H proton peak of ISB observed at 4.40 to 4.61 ppm. *W*_0_ refers to the initial feed mass ratio of silicone.

**Figure 5:**
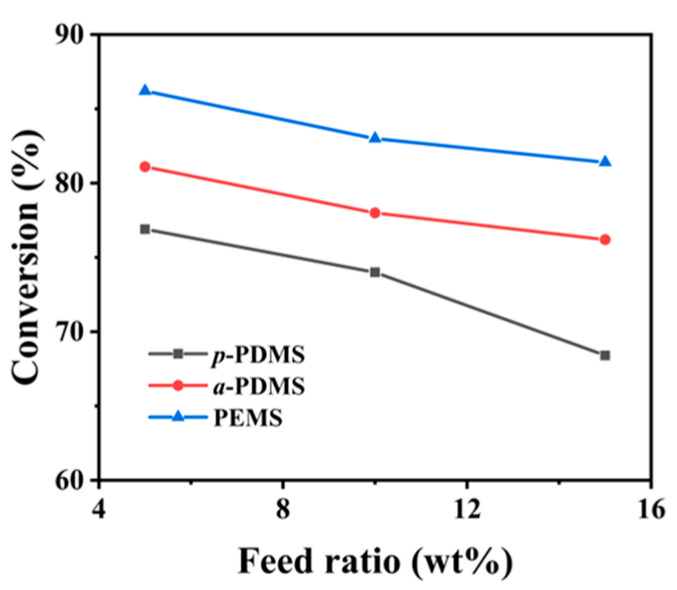
Feed ratio dependence of the silicone conversion.

The conversion rates of three types of silicones with different feed ratios, as shown in Figure 5, indicate a gradual decrease as the amount of silicone feed increases. Moreover, when the amount of silicone feed is the same, the conversion rate of PEMS can reach 86%, higher than that of *a*-PDMS, with *p*-PDMS having the lowest conversion rate. The analysis of the reasons is as follows: Firstly, PEMS exhibits higher reactivity and stronger diffusion ability compared to *a*-PDMS and *p*-PDMS, making it more easily dispersed in the system to participate in reactions. Secondly, due to the polar polyether segments in PEMS, it possesses good water solubility and surface activity, thus having better solubility with hydrophilic ISB and CHDM, increasing system compatibility. Additionally, in the final polycondensation stage, silicone undergoes chain scission under high temperature, vacuum, or strong alkaline catalysis, resulting in the shortening of siloxane chain lengths and the residue of cyclic siloxanes [28,29], which may be the reason why PEMS has trouble reaching conversion rates above 90%.

### 3.4. Physical Property Analysis of ICS-PC

#### 3.4.1. Glass Transition Temperature (*T*_g_)

**Figure 6:**
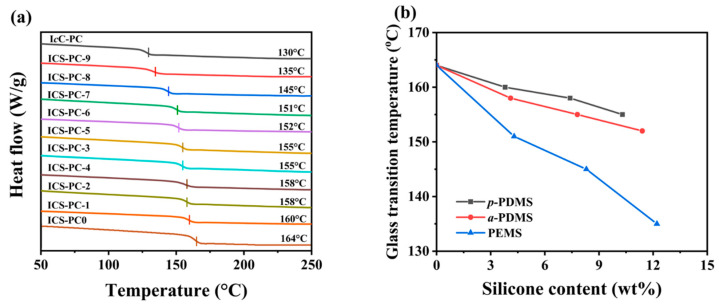
(**a**) DSC curves and (**b**) *T*_g_ vs silicone content for ICS-PC copolymers.

Because of the excellent flexibility of the main chain structure of siloxane, it can enhance the segmental mobility of copolymers and reduce the *T*_g_ of copolymers. Figure 6 shows the DSC spectra of ICS-PCs and the *T*_g_ variation curves. The *T*_g_ of ICS-PC is higher than that of I*c*C-PC with the ISB/CHDM molar ratio of 70/30 because the rigid ISB content increases to 90% in the ICS-PC system. And with the increase in flexible silicone content, the *T*_g_ decreases. Furthermore, the *T*_g_ of the *a*-PDMS system is lower than that of the *p*-PDMS system at the similar silicone content, which may be related to the existence of two rigid phenolic hydroxyl groups in *p*-PDMS monomer, or it may stem from differences in silicone phase size between the two systems. In particular, the *T*_g_ of PEMS system decreases significantly with the increase of silicone content, which may be attributed not only to the addition of low *T*_g_ polyether segments but also to the reduction of PEMS phase size due to the compatibilization of polyether segments, leading to better flexibility. When the PEMS content is 4.3 wt%, the *T*_g_ of copolymer is maintained at 151 °C.

#### 3.4.2. Optical Characterization

**Figure 7:**
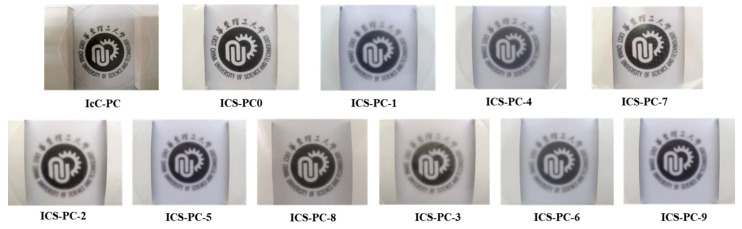
Photos of I*c*C-PC and ICS-PC films with different silicones and contents. (Place the film on a 1 cm platform and compare the transparency of different films based on the clarity of the icons on the desktop.).

Polycarbonate is an optically transparent engineering plastic, but the copolymer exhibits microphase separation after the addition of silicone, resulting in a decrease in light transmittance. Figure 7 shows the photos of various ICS-PC and I*c*C-PC film samples with thicknesses of 80 μm, indicating differences in optical transparency among these samples.

**Figure 8:**
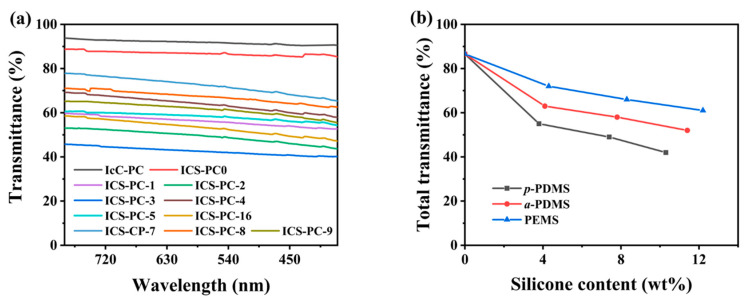
Effect of silicone types and contents on (**a**) light transmission and (**b**) total transmission of copolymer films with thicknesses of 80 μm.

Figure 8 shows the transmittance curves of I*c*C-PC and ICS-PC films in the visible light region. It is observed that the total transmittance of commercially available I*c*C-PC samples is 92%, but the total transmittance of ICS-PC0 synthesized in this study with ISB/CHDM of 90/10 is 87%, attributed to yellowing of the system due to the lack of antioxidants and other additives during the pilot reaction process at high temperatures. The transmittance of ICS-PC films with silicone is lower than that of ICS-PC0 and increases with the decrease in silicone content. However, the total transmittance of both the *a*-PDMS and *p*-PDMS systems does not exceed 63%. In contrast, the transmittance of the PEMS system is higher, with a total transmittance of 72% for ICS-PC-7. The main reason is that the PEMS system exhibits better compatibility during the reaction, with smaller and more uniform dispersed phase sizes, resulting in smaller phase sizes of PEMS in the copolymer and, thus, higher transparency. It is also possible that the refractive index (RI) of PEMS is relatively high, closer to the refractive index of the PC matrix (RI_PC_ = 1.586, RI*_p_*_-PDMS_ = 1.398, RI*_a_*_-PDMS_ = 1.407, RI_PEMS_ = 1.434) [37], thus having a smaller impact on the transparency of the film. Therefore, the PEMS system with higher transmittance provides a new direction for preparing copolymers with high transparency and excellent comprehensive properties. Furthermore, the addition of silicone can hopefully decrease the surface wettability of the copolymer, thus reducing contact with the external environment, prolonging its service life, and enhancing durability.

#### 3.4.3. Hydrophobicity and Water Absorption

**Figure 9:**
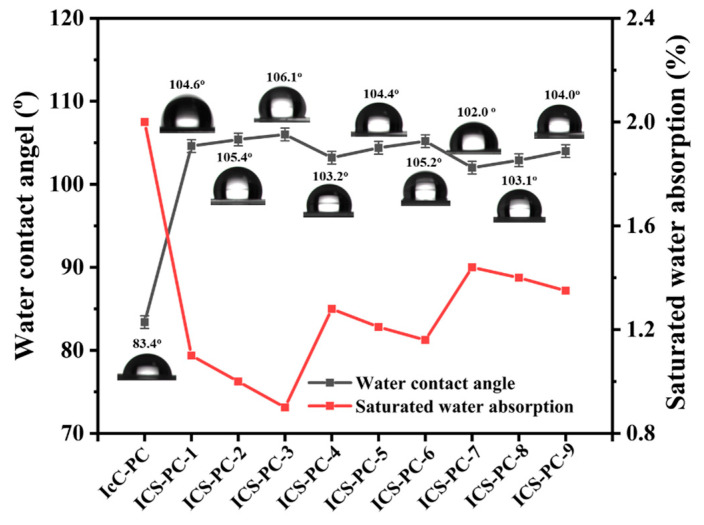
Water contact angle and saturated water absorption of ICS-PC films.

Introducing hydrophobic silicone into PC can reduce the surface energy of the polymer film, thus decreasing its hydrophilicity. Figure 9 shows the water contact angle test results of ICS-PC films with different silicone contents, indicating that as the silicone content increases, the hydrophobic structural units increase, leading to an increase in the water contact angle of the copolymer films. The reason is that compared to the surface tension of PC, which is 40~60 mN/m and water, which is 72 mN/m, the surface tension of silicone is only 20~25 mN/m [38]. Therefore, siloxane segments are prone to aggregate on the film surface due to their low surface tension, enhancing the hydrophobicity of films. Different types of silicone have varying effects on the hydrophobic performance of copolymers. For the *a*-PDMS system, *p*-PDMS, containing a steric hindrance phenyl ring structure, acts as a shield, protecting the internal hydrophilic segments from the influence of external humid environments, resulting in higher water contact angles for its copolymer films [39]. When the *p*-PDMS content is 3.8 wt%, the water contact angle is 104.6°. At similar content, influenced by the hydrophilic polyether segments in the molecular structure, the water contact angle of the PEMS system is 102°, lower than that of the *a*-PDMS and *p*-PDMS systems. Even so, the hydrophobicity of the PEMS system is significantly higher than that of I*c*C-PC. Figure 8 also provides the saturated water absorption of various samples. It is found that the water absorption of ICS-PC is lower than that of I*c*C-PC due to the improved hydrophobicity of copolymers. Although the water absorption of the PEMS system is relatively higher compared to the *a*-PDMS and *p*-PDMS systems, the water absorption of ICS-PC-7 is 1.44%, significantly lower than that of the I*c*C-PC, which is 2%.

#### 3.4.4. Mechanical Properties

**Figure 10:**
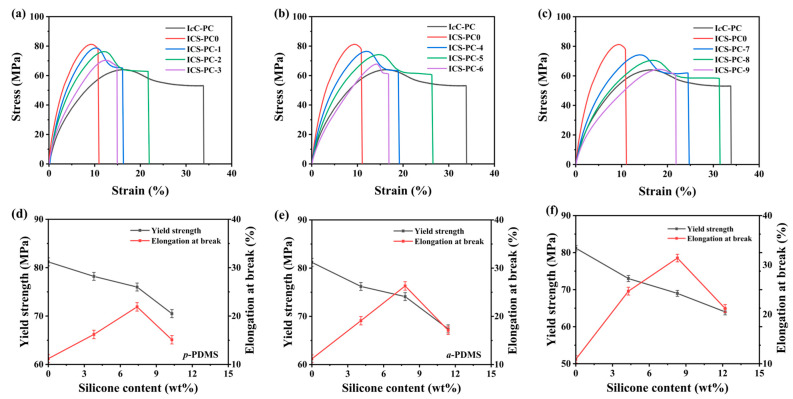
Stress–strain curves for ICS-PCs with (**a**) *p*-PDMS, (**b**) *a*-PDMS, and (**c**) PEMS. Yield strength and elongation at break for ICS-PCs with (**d**) *p*-PDMS, (**e**) *a*-PDMS and (**f**) PEMS.

Figure 10 shows the stress-strain curves of various ICS-PCs and their yield strength and elongation at break. Compared to I*c*C-PC with the ISB/CHDM molar ratio of 70/30, ICS-PC0 has the ISB/CHDM molar ratio of 90/10, where the increased rigid ISB content significantly enhances mechanical strength but decreases elongation at break, making the material brittle. As the silicone content increases, the yield strength of samples gradually decreases while the elongation at break gradually increases. However, when the silicone content exceeds 10 wt%, the elongation at break decreases instead. One reason is that when the silicone content is excessive, partial enrichment of siloxanes leads to an increase in their phase size, resulting in more pronounced microphase separation within the copolymer system. This not only significantly reduces the transparency of the copolymer but also compromises its mechanical properties [40]. Another reason is that the excessive amount of silicone causes the accumulation of siloxane at the surface, disrupting the integrity of the film formation from the solution. It can be seen from the appearance of the film that it becomes turbid and whitish, leading to stress concentration, thereby reducing the strength and toughness of the copolymer. Among the three systems, the *p*-PDMS system exhibits higher mechanical strength, while the PEMS system can play a more flexible role in the system due to the existence of its flexible polyether chain segments, resulting in lower mechanical strength and higher elongation at break for the copolymer. For example, ICS-PC-7 has a yield strength of 74 MPa and an elongation at a break of 25% with good toughness.

## 4. Conclusions

This study investigates the effects of different hydroxy-terminated silicones on the properties of polycarbonate-silicone copolymers (ICS-PC) by introducing flexible hydrophobic monomeric organosilicon into isosorbide-based polycarbonate through melt transesterification-polycondensation. Through compatibility and transesterification experiments, it is confirmed that compared to phenyl-hydroxy silicone (*p*-PDMS) and alcohol-hydroxy silicone (*a*-PDMS), polyether-modified silicone (PEMS) exhibits the highest compatibility and reactivity with other monomers due to the presence of polar polyether segments, achieving a conversion rate of up to 86%. Furthermore, under similar silicone contents, the *p*-PDMS system demonstrates higher *T*_g_, hydrophobicity, and mechanical strength but with a total transmittance of less than 60%, indicating poor optical transparency. In contrast, the PEMS system shows higher optical transparency due to its excellent compatibility with the PC matrix and higher refractive index, achieving a total transmittance of up to 73%, *T*_g_ exceeding 150 °C, as well as excellent toughness and hydrophobicity. These results can serve as a reference for the preparation of high-conversion and high-quality bio-based polycarbonate-silicone copolymers through the melt polycondensation method.

## Figures and Tables

**Table 1:** Effect of silicone types on transesterification reaction.

Silicone	[PhOH] ^1^ (×10^−6^ mol/mL)	[DPC] ^1^ (×10^−6^ mol/mL)	*k*′
PEMS	2.18	0.08	13.33
*a*-PDMS	2.05	0.15	10.24
*p*-PDMS	1.90	0.21	8.00

^1^ Equilibrium concentration of PhOH and DPC.

**Table 2:** Basic properties of ICS-PC.

Sample ^1^	ISB/CHDM	Silicone	Feed Ratio ^2^ (wt%)	*M*_η_ (kg/mol)	*T*_g_ (°C)	Δ*C* (%)	*W*^3^ (wt%)	Conversion ^3^ (%)
IcC-PC	70/30	/	0	35.1	130	0.10	/	/
ICS-PC-0	90/10	/	0	32.8	164	0.11	/	/
ICS-PC-1	90/10	*p*-PDMS	5	31.2	160	0.26	3.8	76.9
ICS-PC-2			10	30.4	158	0.30	7.4	74.0
ICS-PC-3			15	30.0	155	0.35	10.3	68.4
ICS-PC-4	90/10	*a*-PDMS	5	31.4	158	0.24	4.1	81.0
ICS-PC-5			10	31.3	155	0.27	7.8	78.0
ICS-PC-6			15	30.1	152	0.32	11.4	76.2
ICS-PC-7	90/10	PEMS	5	31.2	151	0.19	4.3	86.4
ICS-PC-8			10	30.1	145	0.22	8.3	83.0
ICS-PC-9			15	29.5	135	0.25	12.2	81.4

^1^ All samples were prepared by melt polycondensation using a catalyst of 2 ppm NaOH and a DPC/Diols ratio of 1.01; ^2^ Feeding ratio of silicone; ^3^ The actual content of silicone and its conversion calculated by Equation (8) and Equation (9), respectively.

## Data Availability

Data are contained within the article.

## Supplementary Materials

The following supporting information can be downloaded at https://www.mdpi.com/article/10.3390/polym16101318/s1. Figure S1: ^1^H-NMR spectra of *p*-PDMS and ICS-PC containing *p*-PDMS. Figure S2: ^1^H-NMR spectra of *a*-PDMS and ICS-PC containing *a*-PDMS.

## References

[1] Freitag D., Fengler G., Morbitzer L. (1991). Routes to new aromatic polycarbonates with special material properties. Angew. Chem. Int. Ed..

[2] Hotaka T., Kondo F., Niimi R., Togashi F., Morita Y. (2019). Industrialization of automotive glazing by polycarbonate and hard-coating. Polym. J..

[3] Hoekstra E.J., Catherine S. (2013). Release of bisphenol A from polycarbonate—A review. Crit. Rev. Food Sci. Nutr..

[4] Tsai W.T. (2006). Human health risk on environmental exposure to Bisphenol-A: A review. J. Environ. Sci. Health C. Environ. Carcinog. Ecotoxicol. Rev..

[5] Liu Y., Lu X.B. (2022). Chemical recycling to monomers: Industrial bisphenol-A-polycarbonates to novel aliphatic polycarbonate materials. J. Polym. Sci..

[6] Feng L., Zhu W.X., Li C.C., Guan G.H., Zhang D., Xiao Y.N., Zheng L.C. (2015). A high-molecular-weight and high-*T*_g_ poly(ester carbonate) partially based on isosorbide: Synthesis and structure-property relationships. Polym. Chem..

[7] Wang H., Xu F., Zhang Z.C., Feng M., Jiang M., Zhang S.J. (2023). Bio-based polycarbonates: Progress and prospects. RSC Sustain..

[8] Zenner M.D., Xia Y., Chen J.S., Kessler M.R. (2013). Polyurethanes from isosorbide-based diisocyanates. ChemSusChem.

[9] Medimagh R., Smaidia M.M., Bennour H., Chatti S. (2015). Synthesis and evaluation of the thermal properties of biosourced poly(ether)ureas and copoly(ether)ureas from 1,4:3,6-dianhydrohexitols. Polym. Int..

[10] Chatti S., Schwarz G., Kricheldorf H.R. (2006). Cyclic and noncyclic polycarbonates of isosorbide (1,4:3,6-dianhydro-d-glucitol). Macromolecules.

[11] Fenouillot F., Rousseaua A., Colomines G., Saint-Loup R., Pascault J.P. (2010). Polymers from renewable 1,4:3,6-dianhdrohexitols (isosorbide, isomannide and isoidide): A review. Prog. Polym. Sci..

[12] Thomas J., Pati R.S., John J., Patil M. (2023). A Comprehensive Outlook of Scope within Exterior Automotive Plastic Substrates and Its Coatings. Coatings.

[13] Feng X.H., East A.J., Hammond W.B., Zhang Y., Jaffe M. (2011). Overview of advances in sugar-based polymers. Polym. Adv. Technol..

[14] Nelson A.M., Long T.E. (2012). A perspective on emerging polymer technologies for bisphenol-A replacement. Polym. Int..

[15] Rose M., Palkovits R. (2012). Isosorbide as a renewable platform chemical for versatile applications-quo vadis?. ChemSusChem.

[16] Tundo P., Aricò F., Gauthier G., Rossi L., Rosamilia A.E., Bevinakatti H.S., Sievert R.L., Newman C.P. (2010). Green synthesis of dimethyl isosorbide. ChemSusChem.

[17] Chatti S., Kricheldorf H.R., Schwarz G. (2006). Copolycarbonates of isosorbide and various Diols. J. Polym. Sci. Pol. Chem..

[18] Miyashita M., Yamaguchi M. (2020). Effect of water absorption on the structure and properties of isosorbide-based polycarbonate. Polymer.

[19] Zhou Y.L., Dan Y., Jiang L., Li G.X. (2013). The effect of crystallization on hydrolytic stability of polycarbonate. Polym. Degrad. Stab..

[20] Hein C., Patil R., Zhou B., Lin G.Y., Schmidt C., Zoller D., Ma S., Hassman C. (2018). Polycarbonate Copolymers, Articles Formed Therefrom, and Methods of Manufacture. W.O. Patent.

[21] Chun B., Bahn H., Hwang Y., Park J., Hong M., Lee K., Ko U., Son Y. (2016). Copolycarbonate and Composition Containing Same. W.O. Patent.

[22] Kimura T., Tando K., Oda K. (2019). Polycarbonate-Polydiorganosiloxane Copolymer, Resin Composition of Polycarbonate- Polydiorganosiloxane Copolymer, and Production Method for Resin Composition of Polycarbonate-Polydiorganosiloxane Copolymer. W.O. Patent.

[23] Yilgor I., Yilgor E. (1998). Thermal stabilities of end groups in hydroxyalkyl terminated polydimethylsiloxane oligomers. Polym. Bull..

[24] Zhang S.M., Zhang H.X., Zhang W.Y., Wu Z.Q., Chen F., Fu Q. (2014). Toughening of polycarbonate through reactive melt blending: Effect of hydroxyl content and viscosity of hydroxyl-terminated polydimethysiloxane. Chin. J. Polym. Sci..

[25] Zhou W.J., Osby J. (2010). Siloxane modification of polycarbonate for superior flow and impact toughness. Polymer.

[26] Vaughn H.A. (1965). Organopolysiloxane-Polycarbonate Block Copolymers. U.S. Patent.

[27] Lee J.N., Park C., Whitesides G.M. (2003). Solvent compatibility of poly (dimethylsiloxane)-based microfluidic devices. Anal. Chem..

[28] Malmgren-Hansen B., Olesen S., Pommer K., Funch L.W., Pedersen E. (2003). Survey of Chemical Substances in Consumer Products Survey No. 32—2003 Emission and Evalution of Chemical Substances from Selected Electrical and Electronic Products.

[29] Woodburm K., Drottar K., Domoradzki J., Durham J., Mcnett D., Jezowski R. (2013). Determination of the dietary biomagnification of octamethylcyclotetrasiloxane and decamethylcyclopentasiloxane with the rainbow trout (*Oncorhynchus mykiss*). Chemosphere.

[30] Annett K., Wolfgang E., Walter K. (1998). Preparation of Polysiloxane/Polycarbonate Block Cocondensation Product. J.P. Patent.

[31] Jiyunia J., Makurosuk P., Deibisu G. (1996). Method of Preparing Block Copolysiloxane Carbonate. J.P. Patent.

[32] Koenig A., Ebert W., Koehler W. (1998). Process for the Preparation of Polysiloxane-Polycarbonate Block Cocondensates. D.E. Patent.

[33] Zhou Z.B., Wu G.Z. (2021). Preparation of Bisphenol-A and Polydimethylsiloxane (PDMS) Block Copolycarbonates by Melt Polycondensation: Effects of PDMS Chain Length on Conversion and Miscibility. Polymers.

[34] He M.J., Chen W.X., Dong X.X. (2007). Polymer Physics.

[35] Shinobu Y., Takafumi A., Masami S. (2021). Polycarbonate/Polyorganosiloxane Copolymer and Resin Composition Including Said Copolymer. W.O. Patent.

[36] Yilgor I., Riffle J.S., McGrath J.E. (1985). Reactive Difunctional Siloxane Oligomers.

[37] Venderbosch R.W., Goedmakers J., Hurst J.D. (2005). Translucent Thermoplastic Composition, Method for Making the Composition and Articles Molded Therefrom. U.S. Patent.

[38] Chen X., Lee H.F., Gardella J. (1993). Effects of structure and annealing on the surface composition of multiblock copolymers of bisphenol A polycarbonate and poly(dimethylsiloxane). Macromolecules.

[39] Song Z.Q., Xu F., Wang H., Zhang Z.C., Feng M., Zhang Y.W., Yang Z., Xie J.X., Su D., Li T. (2023). Design and synthesis of isosorbide-based copolycarbonates with high transparency and low hygroscopicity for optical applications. J. Appl. Polym..

[40] Pang X.Y., Ge X., Ji J.Y., Liang W.J., Liu R.L., Chen X.J., Yin G.Q., Ge J.F. (2019). Improving oxygen permeability and thermostability of polycarbonate via copolymerization modification with bio-phenol polysiloxane. Polymers.

