# Supplementary material for: Synthesis and Properties of Bio-Based Polycarbonates Containing Silicone Blocks
Source: Polymers (Basel). 2024 May 8;16(10):1318. doi: 10.3390/polym16101318 (PMC11125172; doi:10.3390/polym16101318)
Supplement: Supplementary file 1 [file polymers-16-01318-s001.zip › polymers-3001945-supplementary.pdf]

Supporting Information for

Synthesis and properties of bio-based polycarbonates containing silicone blocks

Mengjuan Liu, Hui Wang, Wei Fang, Tao Lu, Jinsen Wang and Guozhang Wu *

Shanghai Key Laboratory of Advanced Polymeric Materials, School of Materials Science & Engineering, East China University of Science & Technology, Shanghai 200237, China; 17767145848@163.com (M.L.); y30220861@mail.ecust.edu.cn (H.W.); y82210335@mail.ecust.edu.cn (W.F.); y30210811@mail.ecust.edu.cn (T.L.); y82230330@mail.ecust.edu.cn (J.W.)

* Correspondence: wgz@ecust.edu.cn

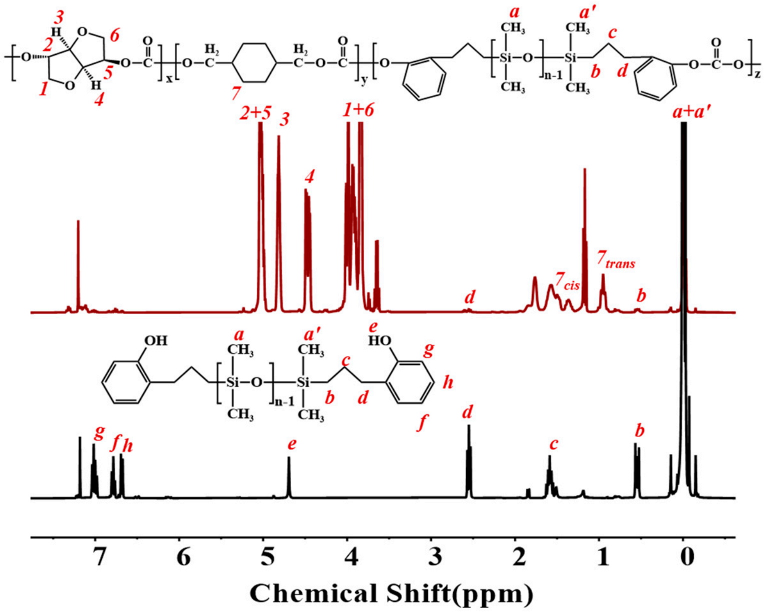

Figure S1. ^{1}H-NMR spectra of *p*-PDMS and ICS-PC containing *p*-PDMS.

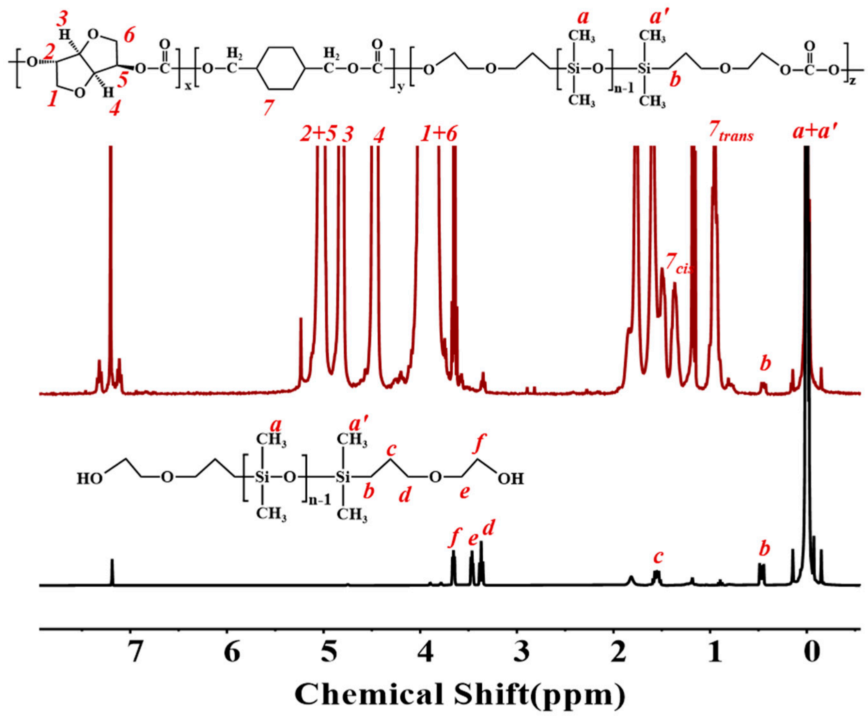

Figure S2. ^{1}H-NMR spectra of *a*-PDMS and ICS-PC containing *a*-PDMS.
